# Comparison of the prevalence of opioid use among U.S. adults with cardiac conditions before and during the COVID-19 pandemic

**DOI:** 10.3389/fpubh.2023.1127636

**Published:** 2023-02-17

**Authors:** Lvkan Weng, Jingxuan Huang, Yanan Lou, Haoting Shi, Yuantong Ma, Siyu Gu, Ne Qiang, Shuxun Wang, Lan Wu, Mu He, Lei Xu, Lefei Han

**Affiliations:** ^1^Shanghai Chest Hospital, Shanghai Jiao Tong University School of Medicine, Shanghai, China; ^2^School of Medicine, Shanghai Jiao Tong University, Shanghai, China; ^3^Immune Therapy Institute, Renji Hospital Affiliated to Shanghai Jiao Tong University School of Medicine, Shanghai, China; ^4^School of Global Health, Chinese Center for Tropical Diseases, Shanghai Jiao Tong University School of Medicine, Shanghai, China; ^5^School of Mathematics and Physics, Xi'an Jiaotong-Liverpool University, Suzhou, China

**Keywords:** COVID-19 pandemic, opioid use, prevalence, survey study, cardiac patients

## Abstract

Limited data are available on the prevalence of prescription opioid use among patients with cardiac conditions who were exposed to increased risks of cardiac events including myocardial failure and cardiac arrest. According to the U.S. National Health Interview Survey, we evaluated the prevalence of opioid use in patients with cardiac conditions who reported prescription opioid use in the past 12 months and 3 months in 2019 and 2020, respectively, and further estimated the prevalence of opioid use for acute pain or chronic pain. We also analyzed the stratified prevalence by demographical characteristics. Our results showed that there was no statistically significant change in the prevalence of opioid use in the past 12 months (26.5% in 2019 vs. 25.7% in 2020) or the past 3 months (66.6% in 2019 vs. 62.5% in 2020) before and during the COVID-19 pandemic. However, there was a significant decline in the prevalence of opioid use for acute pain, from 64.2% (95% confidence interval [CI] 57.6% to 70.3%) in 2019 to 49.6% (95% CI 40.1% to 59.0%) in 2020 (*P* = 0.012), particularly in the subgroups of men, non-Hispanic white people, adults with education below high school, those with an income-to-poverty ratio ranging from 1.0 to 1.9, and those covered with health insurance. Our findings suggest that monitoring opioid use in the era of living with COVID-19 is important, which will help inform healthcare providers to develop care strategies to reduce health loss for vulnerable individuals.

## 1. Introduction

An opioid is the most common analgesic treatment for perioperative, acute, and chronic pain (1). It is recognized as the standard of care for patients with acute coronary syndromes to relieve pain (2) and is also used as an analgesic for those with other cardiovascular diseases (CVDs) (3). However, increasing evidence indicated the cardiotoxic effect of opioid administration (4). There may be an increased risk of endocarditis, hypoxia–ischemia, myocardial failure, and even cardiac arrest with opioids (5). Therefore, it is crucial to direct the safe use of opioids to patients with CVD (6).

Recently, the opioid epidemic has become a public health catastrophe and may worsen due to the COVID-19 pandemic. In 2020, ~70,000 fatal opioid overdoses were recorded in the United States, an increase of ~37% in 2019 (7). However, there are limited data on the prevalence of opioid use among those with cardiac conditions. The lack of timely surveillance may pose challenges for healthcare services providing precise management. Hence, we sought to estimate the prevalence of cardiac patients with opioid use and determine recent trends before and during the COVID-19 pandemic to provide population-scale evidence for the monitoring and management of opioid use.

## 2. Materials and methods

### 2.1. Data source

In this 2-year population-based study, we retrieved data from the National Health Interview Survey (NHIS), which was conducted by the National Center for Health Statistics (NCHS), Center for Disease Prevention and Control of the United States (8). The NHIS is a nationally representatively cross-sectional household survey aimed to surveil health outcomes in civilian non-institutionalized U.S. residents every year. In 2019, the NHIS added new survey content about prescription opioid use and pain management in the sample adult interview (9). In 2020, that information kept being collected as sponsored by the National Center for Injury Prevention and Control. In addition, the NHIS added coronavirus-related content in 2020 (10). The sampling procedure followed a randomized, multistage, and stratified probability approach to recruiting households to collect health-related information by face-to-face or telephone survey. One sample adult from each household was randomly selected to provide his/her health information by himself/herself or a knowledgeable proxy if the sample adults were physically or mentally unable to answer the questionnaire. Through the random and multistage sampling approach, the NHIS created a sample weight for each survey respondent, which conveyed the number of population units each NHIS respondent represents. The sample weights were adjusted for non-response and further adjusted using post-stratification by age, sex, and race/ethnicity based on population estimates from the recent U.S. census information at the time of each NHIS administration, which was computed and provided by the NHIS. The NHIS data were de-identified, publicly available, and approved by the Research Ethics Review Board of the NCHS and the U.S. Office of Management and Budget. This study followed the Strengthening the Reporting of Observational Studies in Epidemiology (STROBE) reporting guidelines.

### 2.2. Data collection

We included sample adults aged 20–79 years with cardiac conditions for analysis. The cardiac conditions were ascertained by asking sample adults whether they ever had coronary heart disease, angina, or heart attack told by doctors. The prescription opioid use of participants was ascertained by asking them whether they have taken any opioid pain relievers prescribed by a doctor, dentist, or other health professionals in the past 3 months and the past 12 months. According to the NHIS criteria, prescription opioid drugs included hydrocodone, Vicodin, Norco, Lortab, oxycodone, OxyContin, Percocet, and Percodan, while over-the-counter pain relievers such as aspirin, Tylenol, Advil, or Aleve were not included (9). The purpose of opioid use (relief of acute pain or chronic pain) was also asked among those who reported taking any opioids prescribed by a doctor in the past 3 months. Opioid use for acute pain was defined as prescription opioid administration to treat short-term or acute pain, such as pain due to a broken bone or muscle sprain, pain from dental work, or pain following surgery, while opioid use for chronic pain was to treat long-term or chronic pain, such as low back pain or neck pain, frequent headaches or migraines, or joint pain or arthritis.

This study included sociodemographic and behavioral characteristics as covariates. Sociodemographic variables included age (grouped into 20–64 years and 65–79 years of age), sex (female and male), race/ethnicity (non-Hispanic white, non-Hispanic Black, Hispanic, and others), educational level (below high school, high school, and beyond high school), income (according to income-to-poverty ratio, IPR, <1.0, 1.0–1.9, 2.0–3.9, and ≥4.0), and health insurance (not covered and covered). The behavioral characteristics included body mass index (BMI stratified into underweight [<18.5 kg/m^2^], normal [18.5–24.9 kg/m^2^], overweight [25.0–29.9 kg/m^2^], and obesity [≥30.0 kg/m^2^]).

### 2.3. Statistical analysis

This study calculated and compared the difference in the prevalence of prescription opioid use in 2019 and 2020. All analyses accounted for the complex weighting variable of the surveys. The sample weights were calculated with adjustment by age, sex, race/ethnicity, educational level, IPR, and BMI and gave prevalence estimates with a 95% confidence interval (CI) for patients with cardiac conditions with opioid use in 2019 and 2020, respectively. Since the outbreak of COVID-19 began in late December 2019 and was declared a global pandemic on 11 March 2020 (11), the prevalence of opioid use in 2019 was considered as the prevalence before the COVID-19 pandemic while that in 2020 was during the COVID-19 pandemic. Student's *t*-test was used to determine the change in the prevalence before and during the COVID-19 pandemic. In addition, to further quantify the impact of the sociodemographic and behavioral variables on the prevalence of opioid use, multivariable logistic regression models were used to calculate the odds ratios (ORs) with the adjustment of age, sex, and race/ethnicity. For all analyses, the level of statistical significance was defined as two-sided *P* < 0.05. The statistical analyses were performed by the R software 4.0.1.

## 3. Results

A total of 4,081 sample adults (*N* = 2,483 in 2019 and *N* = 1,598 in 2020) who disclosed cardiac conditions from a doctor or other health professionals were included in the analyses. Among them, 2,337 (57.3%) adults were men and 1,744 (42.7%) were women. A total of 3,152 (77.2%) were non-Hispanic white, 404 (9.8%) were non-Hispanic Black, 328 (8.0%) were Hispanic, and 197 (4.8%) were other races/ethnicities. There were 1,280 (31.4%) adults aged between 20 and 64 years old, and 2,798 (68.6%) adults aged between 65 and 79 years old. Overall, there were 601 (24.2%) patients with cardiac conditions reporting their use of prescribed opioid drugs in the past 12 months in 2019 and the number was 346 (21.7%) in 2020. The baseline characteristics of participants in 2019 and 2020 are shown in Table 1.

**Table 1:** Characteristics of participants with cardiac conditions in NHIS in 2019 and 2020.

	**No. of participants (%)**		***P*** **-value^a^**
	**2019 (*N* = 2,483)**	**2020 (*N* = 1,598)**	
**Sex**			>0.98
Male	1,421 (57.2)	916 (57.3)	
Female	1,062 (42.8)	682 (42.7)	
**Age, years** ^b^			0.04
20–64	809 (32.6)	471 (29.5)	
≥65	1,672 (67.3)	1,126 (70.5)	
**Race and ethnicity**			0.22
Non-Hispanic White	1,891 (76.2)	1,261 (78.9)	
Non-Hispanic Black	256 (10.3)	148 (9.3)	
Hispanic	212 (8.5)	116 (7.3)	
Others	124 (5.0)	73 (4.6)	
**Education** ^c^			0.007
Below high school	410 (16.5)	207 (13.0)	
High school	738 (29.7)	477 (29.8)	
Beyond high school	1,324 (53.3)	903 (56.5)	
**IPR**			0.01
< 1.0	404 (16.3)	211 (13.2)	
1.0 to 1.9	629 (25.3)	393 (24.6)	
2.0 to 3.9	716 (28.8)	458 (28.7)	
≥4.0	734 (29.6)	536 (33.5)	
Health insurance covered	2,402 (96.7)	1,558 (97.5)	0.19
**BMI** ^d^			0.84
Underweight	26 (1.0)	21 (1.3)	
Normal	579 (23.3)	374 (23.4)	
Overweight	857 (34.5)	567 (33.5)	
Obesity	967 (38.9)	615 (38.5)	
**Cardiac condition**			
Coronary heart disease	1,846 (74.3)	1,224 (76.6)	0.11
Angina	627 (25.3)	378 (23.7)	0.26
Heart attack	1,236 (49.8)	755 (47.2)	0.12
**Opioid use**			
Used in 12 the past months	601 (24.2)	346 (21.7)	0.06
Used in 3 the past months	407 (16.4)	226 (14.1)	0.06
For acute pain, used in the past 3 months	255 (10.3)	116 (7.3)	0.004
For chronic pain, used in the past 3 months	276 (11.0)	157 (9.8)	0.21

IPR, income-to-poverty ratio; BMI, body mass index.

a The differences were examined by the chi-square test, where the missing value was not included in the comparison.

b Age information was missed in two participants in 2019 and one participant in 2020.

c Education information was missed in 11 participants in both 2019 and 2020.

d BMI information was missed in 54 participants in 2019 and 21 participants in 2020. BMI was stratified into underweight (< 18.5), normal (18.5–24.9), overweight (25.0–29.9), and obesity (≥30.0).

The prevalence estimates of prescribed opioid use are shown in Table 2. The estimated prevalence of opioid use in the past 12 months was 26.5% (95% CI 24.0 to 29.2%) in 2019 and 25.7% (95% CI 22.5 to 29.2%) in 2020. No significant difference in the prevalence in 2019 and 2020 was observed (*P* = 0.71). Similarly, the disparities in the prevalence of opioid use within 12 months in 2019 and 2020 by sex, age, race/ethnicity, education, IPR, health insurance, and BMI were statistically non-significant (all *P* > 0.05).

**Table 2:** Comparison of the prevalence of opioid use in 2019 and 2020 by the purpose of use among U.S. adults with cardiac conditions aged 20–79 years.

	**Opioid use, in the past 12 months**			**Opioid use, in the past 3 months**			**Opioid use for acute pain, in the past 3 months**			**Opioid use for chronic pain, in the past 3 months**		
	**Prevalence, %, 2019 (95% CI)**	**Prevalence, %, 2020 (95% CI)**	***P*** **-value ^b^**	**Prevalence, %, 2019 (95% CI)**	**Prevalence, %, 2020 (95% CI)**	***P*** **-value ^b^**	**Prevalence, %, 2019 (95% CI)**	**Prevalence, %, 2020 (95% CI)**	***P*** **-value ^b^**	**Prevalence, %, 2019 (95% CI)**	**Prevalence, %, 2020 (95% CI)**	***P*** **-value ^b^**
Overall	26.5 (24.0, 29.2)	25.7 (22.5, 29.2)	0.712	66.6 (61.3, 71.5)	62.5 (55.2, 69.3)	0.356	64.2 (57.6, 70.3)	49.6 (40.1, 59.0)	0.012	67.9 (60.7, 74.3)	65.3 (55.0, 74.3)	0.666
**Sex**												
Male	23.5 (20.5, 26.8)	23.7 (20.0, 27.8)	0.938	63.6 (56.2, 70.4)	62.7 (53.3, 71.2)	0.877	69.5 (59.4, 78.0)	47.3 (34.7, 60.3)	0.006	68.4 (58.5, 76.9)	62.3 (48.1, 74.7)	0.460
Female	31.2 (27.1, 35.5)	29.2 (23.5, 35.6)	0.595	70.0 (62.9, 76.3)	62.2 (50.3, 72.9)	0.245	58.7 (49.5, 67.2)	52.6 (38.8, 66.1)	0.462	67.4 (57.3, 76.1)	69.3 (53.4, 81.6)	0.826
**Age, years**												
20–64	30.0 (26.0, 34.4)	28.2 (23.3, 33.6)	0.595	71.0 (63.6, 77.5)	63.9 (51.9, 74.3)	0.291	62.3 (52.8, 70.9)	47.0 (34.2, 60.2)	0.058	67.1 (57.1, 75.8)	72.0 (58.6, 82.4)	0.526
65–79	23.2 (20.2, 26.4)	23.8 (19.6, 28.5)	0.828	61.1 (53.4, 68.3)	61.2 (51.7, 70.0)	0.987	67.0 (57.8, 75.0)	52.1 (38.0, 65.9)	0.075	69.0 (59.8, 76.9)	58.6 (43.4, 72.3)	0.225
**Race and ethnicity**												
Non-Hispanic White	26.2 (23.3, 29.3)	27.1 (23.4, 31.0)	0.716	63.6 (57.4, 69.4)	57.7 (50.0, 65.1)	0.230	62.2 (54.0, 69.8)	48.7 (37.9, 59.6)	0.049	68.5 (59.4, 76.4)	63.5 (52.0, 73.6)	0.476
Non-Hispanic Black	29.7 (22.8, 37.7)	26.9 (17.2, 39.6)	0.683	80.4 (65.6, 89.8)	76.3 (38.5, 94.3)	0.792	69.7 (51.8, 83.1)	66.7 (39.8, 85.8)	0.833	58.6 (40.3, 74.8)	55.1 (24.0, 82.7)	0.840
Hispanic	25.4 (19.2, 32.8)	17.7 (8.7, 32.7) ^a^	0.274	63.7 (44.1, 79.5)	78.4 (13.4, 98.8)	0.533	76.8 (55.9, 89.6)	26.9 (0, 100) ^a^	0.064	71.1 (47.6, 86.9)	94.4 (0.0, 100.0)	0.395
Others	26.7 (16.8, 39.7)	18.5 (8.9, 34.5)	0.349	79.8 (38.2, 96.2)	86.9 (36.3, 98.7)	0.744	50.9 (0, 100.0)	44.7 (2.8, 95.8) ^a^	0.859	78.2 (0.0, 100.0)	76.4 (4.0, 99.6)	0.959
**Education**												
Below high school	26.7 (21.3, 33.0)	25.3 (16.7, 36.4)	0.781	68.6 (53.9, 80.3)	62.0 (37.4, 81.7)	0.616	77.1 (62.7, 87.1)	25.9 (5.1, 69.6) ^a^	0.004	67.4 (50.9, 80.5)	60.8 (24.7, 87.9)	0.711
High school	24.4 (19.7, 29.8)	25.2 (18.8, 32.9)	0.811	69.9 (58.4, 79.4)	60.0 (45.6, 72.8)	0.259	53.4 (39.0, 67.3)	53.1 (34.6, 70.7)	0.980	75.3 (62.0, 85.1)	63.6 (42.0, 80.8)	0.310
Beyond high school	27.8 (24.6, 31.4)	26.5 (22.5, 30.9)	0.637	64.2 (57.3, 70.6)	64.3 (54.6, 73.0)	0.986	64.6 (55.9, 72.4)	54.3 (42.1, 66.1)	0.166	64.2 (55.2, 72.3)	67.5 (55.8, 77.3)	0.638
**IPR**												
< 1.0	33.9 (27.4, 41.0)	33.4 (23.9, 44.5)	0.937	81.1 (70.1, 88.7)	55.0 (34.4, 74.1)	0.020	53.1 (39.3, 66.4)	40.6 (17.7, 68.4) ^a^	0.394	82.3 (71.2, 89.7)	67.4 (35.5, 88.6)	0.299
1.0 to 1.9	29.3 (24.1, 35.1)	26.5 (20.1, 34.1)	0.538	70.8 (58.6, 80.6)	68.5 (51.5, 81.7)	0.809	66.2 (53.3, 77.1)	28.0 (15.1, 46.0)	< .001	70.2 (57.4, 80.4)	78.6 (56.7, 91.2)	0.427
2.0 to 3.9	25.4 (21.1, 30.3)	25.0 (19.5, 31.5)	0.917	61.0 (50.6, 70.4)	69.1 (54.1, 80.9)	0.341	75.1 (61.6, 85.1)	54.7 (35.2, 72.8)	0.071	63.5 (48.4, 76.4)	68.9 (47.7, 84.4)	0.647
≥4.0	21.2 (17.4, 25.6)	22.5 (17.9, 27.9)	0.694	55.6 (44.3, 66.3)	56.6 (43.7, 68.7)	0.906	61.6 (45.7, 75.3)	70.2 (47.5, 86.0)	0.488	51.6 (35.3, 67.5)	47.2 (29.3, 65.9)	0.723
**Health insurance**												
Not covered	22.2 (11.8, 37.7)	17.0 (5.3, 43.0) ^a^	0.656	54.7 (16.8, 87.8)	-	-	81.1 (0, 100)	100.0 (N.A, N.A)	-	57.4 (0, 100)	-	-
Covered	26.7 (24.2, 29.4)	26.1 (22.8, 29.6)	0.784	67.0 (61.6, 72.0)	63.5 (56.1, 70.3)	0.436	63.7 (57.0, 69.9)	49.3 (39.8, 58.8)	0.014	68.2 (60.8, 74.8)	65.7 (55.4, 74.7)	0.681
**BMI** ^c^												
Underweight	20.8 (2.8, 70.7) ^a^	21.1 (2.8, 71.6) ^a^	0.990	-	64.7 (0, 100.0) ^a^	-	-	20.5 (0, 100.0) ^a^	-	-	39.0 (0, 100.0) ^a^	-
Normal	25.9 (20.8, 31.8)	22.1 (15.7, 30.1)	0.411	63.7 (50.2, 75.3)	56.3 (35.0, 75.5)	0.543	65.8 (48.2, 79.9)	48.1 (13.8, 84.4)	0.370	81.5 (67.3, 90.4)	72.9 (33.7, 93.4)	0.598
Overweight	24.3 (20.2, 28.9)	23.9 (18.4, 30.4)	0.914	65.6 (55.1, 74.8)	62.8 (49.9, 74.1)	0.725	59.7 (46.8, 71.5)	51.3 (33.4, 68.9)	0.446	62.6 (47.4, 75.7)	52.8 (35.1, 69.7)	0.390
Obesity	28.5 (24.9, 32.4)	28.8 (24.2, 33.8)	0.923	68.5 (61.1, 75.1)	63.3 (52.8, 72.7)	0.402	65.5 (56.3, 73.7)	51.2 (38.4, 63.9)	0.069	67.2 (57.4, 75.6)	71.2 (56.4, 82.5)	0.622

CI, confidence interval; IPR, income-to-poverty ratio; BMI, body mass index; N.A., not available.

a Estimates might be unreliable because of a large relative standard error ≥30%.

b The *P*-value was calculated by Student's *t-*test.

c BMI was stratified into underweight (<18.5), normal (18.5–24.9), overweight (25.0–29.9), and obesity (≥ 30.0).

For the prevalence of opioid use in the past 3 months, there was a non-significant decline, with an estimated value of 66.6% (95% CI: 61.3 to 71.5%) in 2019 and 62.5% (95% CI: 55.2 to 69.3%) in 2020. Subgroup results showed that the decline mainly occurred among patients with IPR of <1.0 in 2020 (prevalence: 81.1% in 2019 vs. 55.0% in 2020, *P* = 0.02), and there was no significant difference in prevalence stratified by age, sex, race/ethnicity, education, health insurance, and BMI between 2019 and 2020 (All *P* > 0.05). In addition, the decline occurred in patients using it for acute pain (Table 2), with an estimate of 64.2% (95% CI: 57.6% to 70.3%) in 2019 and 49.6% (95% CI: 40.1 to 59.0%) in 2020 (*P* = 0.012). Furthermore, the declined prevalence was shown in male subjects (69.5% in 2019 vs. 47.3% in 2020), non-Hispanic white people (62.2% in 2019 vs. 48.7% in 2020), those with an education level below high school (77.1% in 2019 vs. 25.9% in 2020), those with IPR from 1.0 to 1.9 (66.2% in 2019 vs. 28.0% in 2020), and those with covered health insurance (63.7% in 2019 vs. 49.3% in 2020). In contrast, there was no significant change in opioid use for chronic pain with an estimated prevalence of 67.9% (95% CI: 60.7 to 74.3%) in 2019 and 65.3% (95% CI: 55.0 to 74.3%) in 2020 (*P* > 0.05).

According to the results from multivariable logistic regression models, we found that family income level might be associated with opioid use in patients with cardiac conditions (Table 3). Opioid use within 12 months was less prevalent among adults with higher family income levels (for IPR≥ 4.0, OR 0.57 [95% CI: 0.38 to 0.85] in 2019; 0.54 [95% CI: 0.32 to 0.94] in 2020). Similarly, in 2019, the prevalence of opioid use within 3 months and opioid use for chronic pain were lower among higher family income levels; however, this effect vanished in 2020. Notably, those with higher family income levels were prone to use opioids for acute pain (for IPR 2.0 to 3.9, OR 2.45 [95% CI: 1.01 to 5.94] in 2019, 3.22 [95% CI: 1.01 to 10.32] in 2020; for IPR ≥4.0, OR 1.35 [95% CI: 0.53 to 3.45] in 2019, 7.77 [95% CI: 2.10 to 28.70]). In addition, the prevalence of opioid use for acute pain was positively associated with high education in 2020 (OR 5.38 [95% CI: 1.48 to 19.6] for adults in high school and OR 5.58 [95% CI: 1.63 to 19.11] for adults beyond high school, respectively), though non-significant or negatively in 2019. We also found that the prevalence was higher among those aged 65–79 years than those aged 20–64 years old in 2019 and not found in 2020 or other subgroups.

**Table 3:** Adjusted odds ratios of opioid use by subgroup and purpose of use before in 2019 and 2020 among US adults with cardiac conditions aged 20–79 years.

	**Opioid use, in the past 12 months**		**Opioid use, in the past 3 months**		**Opioid use for acute pain, in the past 3 months**		**Opioid use for chronic pain, in the past 3 months**	
	**Adjusted OR, 2019 (95% CI)**	**Adjusted OR, 2020 (95% CI)**	**Adjusted OR, 2019 (95% CI)**	**Adjusted OR, 2020 (95% CI)**	**Adjusted OR, 2019 (95% CI)**	**Adjusted OR, 2020 (95% CI)**	**Adjusted OR, 2019 (95% CI)**	**Adjusted OR, 2020 (95% CI)**
**Sex**								
Male	Ref	Ref	Ref	Ref	Ref	Ref	Ref	Ref
Female	0.71 (0.54, 0.93)	1.36 (0.93, 1.97)	1.25 (0.81, 1.93)	0.94 (0.51, 1.73)	0.61 (0.34, 1.07)	1.16 (0.54, 2.49)	0.97 (0.53, 1.78)	1.52 (0.64, 3.61)
**Age, years**								
20–64	Ref	Ref	Ref	Ref	Ref	Ref	Ref	Ref
≥65	1.46 (1.12, 1.91)	0.75 (0.52, 1.09)	0.69 (0.43, 1.10)	1.00 (0.53, 1.92)	1.21 (0.68, 2.14)	1.09 (0.51, 2.33)	1.03 (0.58, 1.82)	0.62 (0.28, 1.37)
**Race and ethnicity**								
Non-Hispanic White	Ref	Ref	Ref	Ref	Ref	Ref	Ref	Ref
Non-Hispanic Black	1.06 (0.73, 1.54)	0.93 (0.50, 1.72)	2.10 (0.96, 4.59)	2.38 (0.57, 9.87)	1.62 (0.74, 3.55)	2.07 (0.73, 5.90)	0.66 (0.28, 1.56)	0.74 (0.23, 2.35)
Hispanic	0.90 (0.60, 1.34)	0.54 (0.23, 1.27)	1.08 (0.50, 2.31)	2.64 (0.59, 11.80)	1.97 (0.79, 4.90)	0.41 (0.07, 2.47)	1.12 (0.42, 2.96)	10.10 (0.96, 106.04)
Others	0.95 (0.53, 1.73)	0.58 (0.26, 1.29)	2.11 (0.66, 6.74)	4.91 (0.83, 29.09)	0.73 (0.24, 2.24)	0.88 (0.18, 4.26)	1.67 (0.44, 6.33)	1.55 (0.17, 14.45)
**Education**								
Below high school	Ref	Ref	Ref	Ref	Ref	Ref	Ref	Ref
High school	0.84 (0.56, 1.27)	0.90 (0.48, 1.69)	0.95 (0.43, 2.09)	1.14 (0.41, 3.23)	0.34 (0.14, 0.81)	5.38 (1.48, 19.60)	0.48 (0.21, 1.10)	1.10 (0.30, 40.0)
Beyond high school	1.04 (0.72, 1.49)	0.97 (0.55, 1.73)	0.73 (0.38, 1.39)	1.38 (0.51, 3.75)	0.56 (0.26, 1.20)	5.58 (1.63, 19.11)	0.87 (0.41, 1.85)	1.40 (0.44, 4.51)
**IPR**								
<1.0	Ref	Ref	Ref	Ref	Ref	Ref	Ref	Ref
1.0 to 1.9	0.84 (0.56, 1.27)	0.73 (0.41, 1.28)	0.61 (0.27, 1.37)	2.06 (0.75, 5.69)	1.73 (0.79, 3.82)	0.85 (0.26, 2.84)	0.48 (0.21, 1.10)	1.44 (0.32, 6.46)
2.0 to 3.9	0.72 (0.49, 1.05)	0.65 (0.38, 1.11)	0.41 (0.19, 0.86)	2.54 (0.92, 7.01)	2.45 (1.01, 5.94)	3.22 (1.01, 10.32)	0.34 (0.14, 0.82)	0.75 (0.21, 2.74)
≥4.0	0.57 (0.38, 0.85)	0.54 (0.32, 0.94)	0.33 (0.15, 0.72)	1.66 (0.65, 4.28)	1.35 (0.53, 3.45)	7.77 (2.10, 28.71)	0.19 (0.07, 0.48)	0.34 (0.08, 1.38)
**Health insurance**								
Not covered	Ref	Ref	Ref	Ref	Ref	Ref	Ref	Ref
Covered	1.49 (0.72, 3.09)	1.69 (0.53, 5.38)	2.64 (0.81, 8.62)	9.65 (1.24, 75.10)	0.40 (0.04, 3.62)	-	1.25 (0.23, 6.86)	-
**BMI** ^a^								
Underweight	Ref	Ref	Ref	Ref	Ref	Ref	Ref	Ref
Normal	1.53 (0.39, 6.00)	1.26 (0.29, 5.54)	0.71 (0.05, 10.14)	0.51 (0.04, 6.15)	0.73 (0.04, 15.07)	3.85 (0.25, 59.50)	0.48 (0.21, 1.10)	1.44 (0.32, 6.46)
Overweight	1.46 (0.39, 5.52)	1.37 (0.30, 6.17)	0.89 (0.07, 11.64)	0.83 (0.07, 9.66)	0.52 (0.03, 10.36)	3.89 (0.27, 56.46)	3.43 (0.19, 63.29)	2.17 (0.18, 25.41)
Obesity	1.69 (0.45, 6.37)	1.70 (0.39, 7.46)	0.98 (0.07, 12.87)	0.75 (0.07, 8.33)	0.68 (0.03, 13.30)	3.76 (0.27, 52.53)	4.22 (0.24, 73.06)	4.99 (0.43, 58.26)

OR, odds ratio; IPR, income-to-poverty ratio; BMI, body mass index; Ref, reference group.

a BMI was stratified into underweight (<18.5), normal (18.5–24.9), overweight (25.0–29.9), and obesity (≥30.0).

## 4. Discussion

In this study, we used nationally representative data from the population-based NHIS to estimate the prevalence of prescription opioid use in patients with cardiac conditions before and during the COVID-19 pandemic. We also analyzed the stratified prevalence by sociodemographic and behavioral characteristics and the purpose of use for acute pain or chronic pain relief. We did not find a significant change in the prevalence of opioid use before and during the COVID-19 pandemic. However, a decreased prevalence of opioid use in the past 3 months was observed for acute pain, particularly in the subgroups of men, non-Hispanic whites, adults with an education level below high school, those with IPRs ranging from 1.0 to 1.9, and those covered with health insurance.

To the best of our knowledge, this is the first nationally representative study to estimate the prevalence of prescription opioid use in patients with cardiac conditions. Previous studies reported that there were 34% of civilian, non-institutionalized adults in the United States reported having used at least one of these specific prescription opioids at least once in the past 12 months, according to the 2015 National Survey on Drug Use and Health (NSDUH) questionnaire items (12). However, our study reported a prevalence of approximately 26% of opioid use with cardiac conditions, lower than estimates in previous years among the general population, although patients with cardiac conditions are thought to potentially have more opioid use. Differences in study design, sampling approaches, data collection procedure, and participant characteristics may partly explain the prevalence differences. The differences between NSDUH and NHIS had been reported previously (13). Moreover, the expanded definition of opioid use might also be the reason. In NHIS, all the opioid use was followed by a doctor, dentist, or other health professionals while not in NSDUH.

Emerging evidence indicated that the COVID-19 pandemic would result in significant increases in opioid use (14, 15). However, no significant changes were observed in our study. This may be due to the following reasons. First, patients with cardiac conditions may be in more careful management, as a result of which the use of opioids may be more regulated. In addition, with restrictions on face-to-face clinical consultations during the COVID-19 pandemic, prescription opioids were more difficult to obtain, which also partly explained the decline in the prevalence of opioid use for acute pain since the COVID-19 pandemic.

The association between income and opioid use was reported previously (16). Consistent with our results, individuals with lower income had a higher level of exposure than those with higher income to opioid prescriptions, though the racial and ethnic disparities were not observed, which might be due to the better management of cardiac events and better health awareness among patients with high income (17, 18). However, the fact that those with higher incomes were prone to use opioids for acute pain has not been reported before, particularly during the COVID-19 pandemic. This might be because patients with high incomes were more able to get opioid prescriptions. It was documented that patients with high incomes were more likely to have access to healthcare during the COVID-19 pandemic (19). In addition, clinicians were more likely to prescribe opioids for pain management to white patients than to racial/ethnic minority patients presenting with the same symptoms (20, 21), which might also exist in high-income vs. low-income patients. Therefore, more studies were warranted to further describe the association between opioid use and income.

Some limitations should be noted in this study. First, the cardiovascular conditions from the NHIS data were confirmed by self-report or proxy report, which may be subjected to recall bias and lead to misclassification of individuals who have heart conditions. Second, NHIS data did not provide additional information about the purpose of opioid use. It is unclear to further understand whether the drugs were used for cardiac events or other purposes. Third, due to the COVID-19 pandemic, the face-to-face survey was hard to achieve and switched to telephone surveys, leading to a decline in survey response rates. Our results should be carefully interpreted in case of the low response disproportionately occurred in particular populations.

## 5. Conclusion

This study provides national prevalence estimates on opioid use in U.S. patients with cardiac conditions before and during the COVID-19 pandemic. Although the overall prevalence of opioid use among patients with cardiac conditions in 2019 and 2020 leveled off, there was a decline in the prevalence of opioid use in 2020 among the cardiovascular populations who reported using prescription opioids in the past 3 months to relieve acute pain. As the COVID-19 pandemic may continue posing health threats and changing normal life, it is important to keep monitoring opioid use among vulnerable populations. Further investigations are in need to understand the factors associated with the change in opioid use among patients with cardiac conditions in the era of living with COVID-19.

## Data availability statement

Publicly available datasets were analyzed in this study. This data can be found here: https://www.cdc.gov/nchs/nhis/data-questionnaires-documentation.htm.

## Author contributions

LWe conceived and designed the study. LWe, JH, and HS acquired the data. JH, HS, SG, and NQ cleaned and analyzed the data. JH and YL interpreted the results. LWu and JH drafted the manuscript. LWe, JH, YL, HS, YM, SG, NQ, SW, LWu, MH, LX, and LH revised the manuscript. All authors contributed to the content and critical revision of the manuscript and approved the final version.
